# The Hospitals and Dispensaries of Burma

**Published:** 1904-01-02

**Authors:** 


### THE HOSPITALS AND DISPENSARIES OF BURMA.

THE triennial report for the years 1899-1901 shows that the number of hospitals and dispensaries under Government supervision in Burma on January 1st, 1901, was 115, and that during the year six new dispensaries had been established and two closed. But the ratio per cent. of patients treated in respect to the total of population had only been 10·08, demonstrating that as yet only the fringe of the people has been touched by the medical charities of the province. However, there appears to be a laudable desire on the part of district officials to bring medical relief within easy reach of the people, for in reply to a circular letter on the desirability of establishing dispensaries at every township headquarters applications to open 102 new dispensaries were received. At present, owing to the regrettable paucity of hospital assistants and the difficulty of recruiting men of this class for Burma, the Government can do nothing towards granting these requests. As a whole, the subordinate Medical Department has done, and continues to do, good and useful work, more especially when the workers have been in the military department where they learnt subordination and discipline. But some of the men, notably the Bengali, are reported as not possessing the slightest sense of duty, invariably scamping their work, and even malingering so as to shirk an unpleasant duty or get away from a climate which they do not like. The number of patients treated in the three years ending 1901 was 2,576,913, or an increase of 280,343 over the total of the three previous years ending 1898. But full advantage is not taken of the beds provided for indoor patients. Even those who do enter the hospitals are many of them travelling mendicants, not by any means unwishably ill, but just sufficiently sick to allow them to occupy beds which would otherwise remain empty. Of these in-patients, for the year 1901, 89 per cent. are males, 9 per cent. females, and 2 per cent. children. The small number of women and children desiring hospital treatment is apparently due to the fact that the local Burman does not willingly resort to hospitals for indoor treatment, preferring his own house for his family when sick, and that the natives of India, who largely fill the male wards, are not often accompanied to Burma by their womenkind. The increase in the number of outdoor patients is satisfactory, and this is partly because considerable improvements have been made during the year in connection with the comforts and conveniences of patients, such as separate waiting accommodation, and separate dressing-rooms for males and females; a better supply of douches and fittings; more attention to aseptic precautions on the part of the dresser, and to the engagement of a female nurse wherever funds are available. Everywhere the municipal authorities have been urged to provide a Dufferin nurse to attend upon the women patients, both indoor and out, and 15 nurses are now being employed with permission to engage also in private practice as midwives. There has been a steady increase in surgical operations at the General Hospital, Rangoon, during the past three years, and chloroform was administered 1,076 times without any accident. Out of 416 cases of beriberi treated in this hospital only 219 died, and special investigations have been made on the subject. Some medical men believe that beriberi is attributable to diseased rice, but the illness is rare among Burmans, who are essentially rice eaters. The only thing which is said to be certain is that it is disease of locality, and that those who reside upon the light-ships off the coast are especially liable after about three months' residence. If removed in time from the locality where the disease has started, and given fresh food, the patient can generally be cured, but all theories as to the cause are at present in need of proof.

